# Development and Techno-Economic Evaluation of Crystallization Techniques for GABA Purification from Fermentation Broth

**DOI:** 10.3390/molecules30040897

**Published:** 2025-02-14

**Authors:** Yu Jing, Jinxu Zhang, Shengping You, Mengfan Wang, Rongxin Su, Wei Qi

**Affiliations:** 1 Chemical Engineering Research Center, School of Chemical Engineering and Technology, State Key Laboratory of Chemical Engineering, Tianjin University, Tianjin 300350, China; jy_waldeinsamkeit@163.com (Y.J.); 13582364360@163.com (J.Z.); surx@tju.edu.cn (R.S.); qiwei@tju.edu.cn (W.Q.); 2 School of Life Sciences, Tianjin University, 92 Weijin Road, Nankai District, Tianjin 300072, China; mwang@tju.edu.cn; 3 State Key Laboratory of Chemical Engineering, Tianjin University, Tianjin 300072, China

**Keywords:** γ-aminobutyric acid, crystallization, purification, techno-economic analysis

## Abstract

γ-aminobutyric acid, a critical neurotransmitter, is experiencing an increasing demand in the medical and health fields. Conventional processes for γ-aminobutyric acid purification from fermentation broth encounter significant challenges, such as high ethanol usage, low yield, complex process flow, and environmental pollution. Therefore, a purification process based on crystallization techniques was developed to address the above issues. The process was implemented in two stages: desalination and γ-aminobutyric acid treatment. Na_2_SO_4_ was effectively removed through a cooling crystallization technique. γ-aminobutyric acid with a purity of 98.66% and a yield of 67.32% was further obtained through a designed “antisolvent-cooling” crystallization process in a 3.2 L system. Moreover, the new process reduced ethanol usage compared to conventional processes, streamlined the purification process flow, and was more environmentally sustainable. Furthermore, we established an industrial-scale model for γ-aminobutyric acid production. Techno-economic analysis indicates that an investment in a plant with an annual capacity of 74.16 tons of γ-aminobutyric acid is projected to achieve payback in 1.98 years. In conclusion, the crystallization-based purification process is poised for industrial-scale γ-aminobutyric acid production due to its high efficiency, economic viability, energy conservation, and environmental compatibility.

## 1. Introduction

γ-aminobutyric acid, a four-carbon non-protein amino acid, is extensively present across various organisms [1,2,3]. γ-aminobutyric acid serves as the primary inhibitory neurotransmitter in the mammalian central nervous system, playing a pivotal role in neuromodulation and numerous physiological functions [4]. Its therapeutic potential extends to treating neurological disorders [5,6,7], alleviating psychiatric symptoms [8,9], regulating blood pressure and heart rate [10,11], promoting sleep quality [12], delaying aging processes [13], and so on. Recent studies have highlighted the potential application of γ-aminobutyric acid in cancer therapy [14]. Consequently, the demand for γ-aminobutyric acid applications in medicine and health fields is rapidly increasing.

γ-aminobutyric acid can be obtained by chemical synthesis [15], plant extraction and enrichment [16,17,18], or microbial fermentation processes [19,20]. The plant extraction method poses limitations for large-scale industrial production due to challenges associated with extraction efficiency and low yield. Chemical synthesis is unsuitable for producing γ-aminobutyric acid for food or medicinal applications because of harsh conditions and the generation of harmful byproducts. In contrast, microbial fermentation stands out as the preferred method due to its environmentally friendly nature, high efficiency and selectivity, high yield, and safety [1]. Nevertheless, the complex composition of fermentation broth brings considerable challenges to downstream purification processes.

The purification of γ-aminobutyric acid from fermentation broth typically involves four stages: flocculation, decolorization, desalination, and γ-aminobutyric acid treatment [21]. Notably, the desalination and γ-aminobutyric acid treatment stages are the focal points of research interest, as they directly influence the purity and yield of γ-aminobutyric acid. Ethanol desalination, originally developed by Li et al. [21], is a commonly used desalination method. The method is based on the principle that Na_2_SO_4_ is insoluble in 70% ethanol, while γ-aminobutyric acid remains soluble in this solution. However, this method has several notable drawbacks. Firstly, it consumes substantial ethanol as a reagent; secondly, the removal of ethanol through rotary evaporation is energy-intensive. More importantly, the desalination efficiency is restricted due to concerns over γ-aminobutyric acid loss. Common methods for γ-aminobutyric acid treatment such as ion exchange [21,22] and ethanol precipitation [23] also have inherent limitations. Ion exchange resins are costly and operationally demanding, with frequent replacements due to diminishing exchange capacity. Additionally, their pretreatment and regeneration consume significant amounts of acid and alkali, generating considerable wastewater and posing environmental concerns. Importantly, ammonia cannot be used as an eluent for the purification of food-grade γ-aminobutyric acid. The ethanol precipitation method is inefficient, with high ethanol usage and low γ-aminobutyric acid yields. Additionally, it requires ethanol recovery and filtrate concentration for a secondary precipitation step to increase γ-aminobutyric acid yield, which is energy-intensive and impractical for industrial-scale applications. In summary, current γ-aminobutyric acid purification processes have several limitations, highlighting an urgent requirement for the development of a green, environmentally friendly, and energy-efficient purification process.

Here, this study developed a crystallization-based purification process for γ-aminobutyric acid focusing on desalination and γ-aminobutyric acid treatment stages. The cooling crystallization technique was employed to remove Na_2_SO_4_. The effects of various factors on the removal efficiency were systematically investigated to identify optimal cooling crystallization conditions. Subsequently, a coupled crystallization process of “antisolvent-cooling” was designed and optimized. Then, we applied the integrated crystallization process to a 3.2 L system. Finally, we established an industrial production process model for γ-aminobutyric acid and conducted a techno-economic analysis. This research offers an innovative solution to the issues of high ethanol usage, energy consumption, complex process flow, environmental pollution, and low γ-aminobutyric acid yields associated with traditional γ-aminobutyric acid purification methods.

## 2. Results and Discussion

### 2.1. Optimization of Cooling Crystallization Desalination

This study initially assessed the feasibility of the cooling crystallization desalination method by measuring the solubility of γ-aminobutyric acid (GABA; subsequently, the abbreviation GABA is used to represent γ-aminobutyric acid) and Na_2_SO_4_ in water, as well as Na_2_SO_4_ in solutions with varying GABA concentrations (Figure S2). The experiment was subsequently conducted using a prepared GABA-Na_2_SO_4_ solution to eliminate interference from impurities in the fermentation broth. Preliminary single-factor experiments pinpointed that the crystallization terminal temperature, crystallization time, and stirring rate are critical operational parameters in the cooling crystallization process (Figure S3). In addition to the individual effects of the factors, the response variables are also influenced by the interactions among them. RSM provides a clear framework for assessing the effects of multiple factors. The outcomes of our experimental design were analyzed via quadratic multiple regression utilizing Design-Expert 13 software, followed by predictions of removal efficiency conducted through analysis of variance (ANOVA). The quadratic regression equation relating the response variable R to each influencing factor is outlined as follows:

R = 72.25 − 3.94A + 0.7340B + 2.38C − 0.4000AB − 0.3850AC + 0.3500BC − 8.48A^2^ − 3.83B^2^ − 2.21C^2^ (1)

where R is the removal efficiency (%); A, B, and C represent the crystallization terminal temperature (°C), stirring rate (r/min), and crystallization time (h), respectively.

Table 1 presents the results of RSM analysis of variance. The F value is 40.80, with a significance level of *p* < 0.0001, indicating that the quadratic model is highly significant (*p* < 0.01). In addition, the high R² value of 0.9735, along with an adjusted R² of 0.9496 and a predicted R² of 0.8723, indicates that the model fits well to the data. The “Adequate Precision” was employed to assess the signal-to-noise ratio, with an ideal value of over 4 [24]. The Adequate Precision for this design is calculated at 17.3848, which indicates a robust signal strength, confirming that the model can effectively predict the removal efficiency.

**Figure 1:**
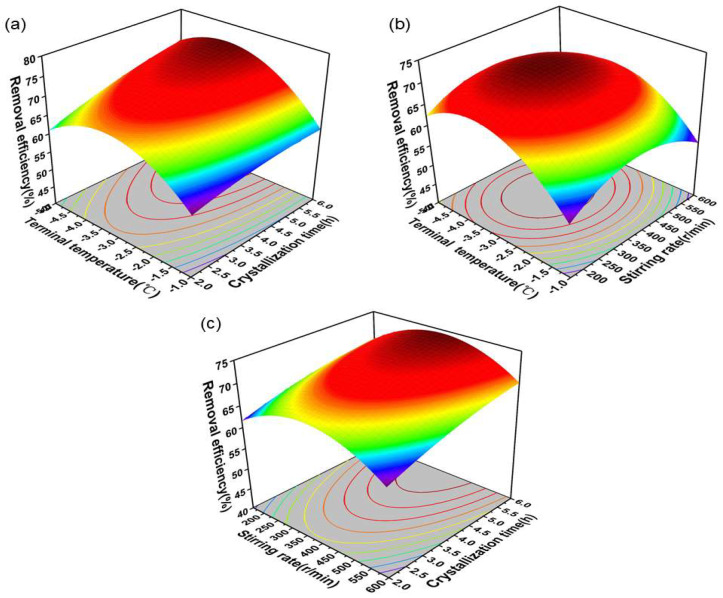
Three-dimensional response surface curve of interaction of different factors on removal efficiency: (**a**) effect of crystallization terminal temperature and crystallization time; (**b**) effect of crystallization terminal temperature and stirring rate; (**c**) effect of stirring rate and crystallization time.

The three-dimensional (3D) response surface curves illustrate the interaction between two distinct process parameters affecting the removal efficiency (Figure 1), while all other variables in the graph are maintained at their median operating levels. The main findings can be summarized as follows: Although the decrease in the crystallization terminal temperature and increase in the crystallization time exhibited a significant enhancement in the removal efficiency, the effect of crystallization terminal temperature was notably more pronounced. The effect of stirring rate appeared less significant. This can be attributed to its primary role in facilitating heat and mass transfer within the crystalline system. Even at lower stirring rates, a longer crystallization time tended to render the system homogeneous, which led to a reduced influence of stirring rate compared to other operational factors. Alongside the F and *p* values presented in Table 1, these observations enabled us to rank the effects of critical parameters on measured responses in the following order: crystallization terminal temperature > crystallization time > stirring rate. Furthermore, the convexity observed in the variation of removal efficiency across each three-dimensional response surface indicated that maximum removal efficiency can be attained at the highest levels of independent variables. The optimized regression model forecasts a removal efficiency of 73.48% under the conditions of a crystallization terminal temperature of −3.49 °C, crystallization time of 5.14 h, and stirring rate of 409.63 r/min. The experimental result obtained was 73.15%, which closely corresponds to the predicted value, substantiating the applicability of the model. Initially, the removal efficiency recorded was 65.56%, while the optimized rate reached 73.15%. In conclusion, RSM effectively identified optimal process parameters for enhanced desalination.

Through the optimization of parameters, the primary cooling crystallization attained a removal efficiency of 73.15%, while the purity of the GABA product obtained was limited to 94.47%. This phenomenon can be attributed to the extremely low solubility of Na_2_SO_4_ in ethanol. During the “antisolvent-cooling” crystallization process in GABA production, residual Na_2_SO_4_ precipitated from the mother liquor, thereby reducing product purity. Consequently, enhancing removal efficiency is imperative. Following the second desalination treatment, the removal efficiency increased to 90.79%, and GABA purity notably increased to 99.4%, indicating that a higher removal efficiency is conducive to GABA purity enhancement. Therefore, two desalination processes were carried out by cooling crystallization.

### 2.2. Optimization of the Process Conditions of “Antisolvent–Cooling” Coupled Crystallization

**Figure 2:**
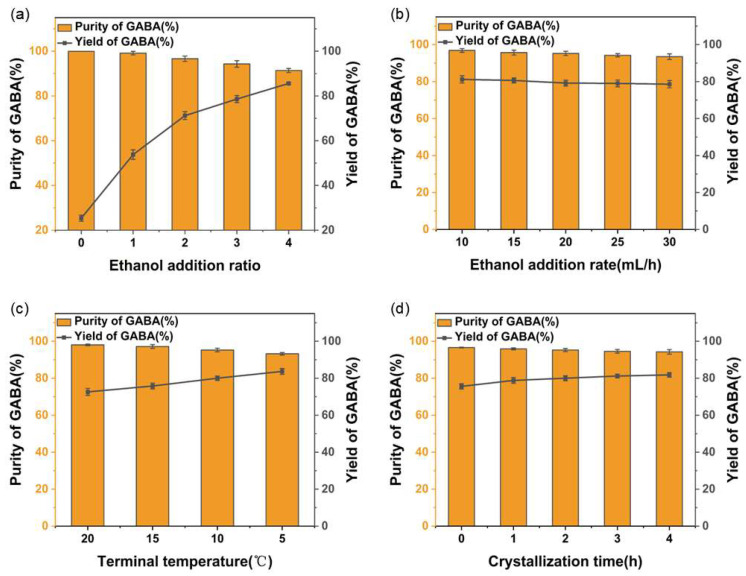
Optimization of crystallization process: (**a**) ethanol addition ratio; (**b**) ethanol addition rate; (**c**) terminal temperature; (**d**) crystallization time.

Given the high aqueous solubility of GABA and its minimal temperature dependence, yields from cooling or evaporation alone are significantly low. Therefore, this study designed a coupled crystallization process of “antisolvent–cooling”. Ethanol was added as the antisolvent to decrease the solubility of GABA in solution, while dilution of the system enhanced mass and heat transfer efficiency. This approach was synergized with cooling to facilitate GABA crystallization. The results are illustrated in Figure 2.

The amount of ethanol added signifi”antl’ ©nfluences the crystallization process (Figure 2a). In the absence of ethanol, the GABA yield was limited to 25.36%, thereby confirming the necessity of ethanol as an antisolvent. As the amount of ethanol added increased, the yield of GABA tended to rise, while the purity gradually decreased. This could be attributed to the increase of ethanol addition promoting the precipitation of other impurities, thereby affecting the purity of GABA. When three-times the volume of concentrate in ethanol was added, GABA purity and yield reached 94.33% and 78.6%. The investigation into the ethanol addition rate (Figure 2b) indicated that an increase in the addition rate results in a decrease in GABA purity. Considering time efficiency, the optimal rate was identified as 20 mL/h. The reduction in temperature aimed to decrease GABA solubility within the crystallization system. Notably, lower terminal temperatures (Figure 2c) were correlated with enhanced yields, but excessively low temperatures precipitated additional impurities that compromised overall purity levels. To achieve an equilibrium between both purity and yield, a cooling terminal temperature set at 10 °C was selected. Additionally, exploring various durations for crystallization (Figure 2d) indicated that prolonged crystallization time negatively impacted GABA purity while exerting minimal influence on yield. An ideal crystallization time was determined to be 2 h. Therefore, final parameters for the “antisolvent–cooling” crystallization process were established as follows: an ethanol addition ratio of 3:1, a rate of ethanol addition at 20 mL/h, a crystallization terminal temperature maintained at 10 °C, and a crystallization time of 2 h. These conditions led to GABA purity and yield reaching up to 95.26% and 80%, respectively. To further improve the purity of GABA, the harvested crystals underwent a recrystallization process. Optimal conditions resulted in a GABA purity of 98.73%, with a total yield of 76.45% across two crystallization processes.

### 2.3. Scale-Up Test of Purification of GABA from Fermentation Broth

Based on the previously optimized GABA purification methods, this study scaled up the process to 3.2 L to explore the effectiveness and feasibility of crystallization process at a larger operational scale. The experimental results are shown in Table 2. In the 3.2 L system, the simulated fermentation solution achieved a removal efficiency of 92.58% after cooling crystallization, which shows an improvement compared to small-scale experiments. Meanwhile, the GABA yield in the actual fermentation broth increased to 67.32%. This result is attributed to the good scalability of the crystallization process in large-scale operations [25]. Additionally, the purity of GABA was 98.66%, maintaining a high standard, which provides a strong guarantee for subsequent large-scale production and application. In conclusion, the findings of this study suggest that the GABA crystallization purification method employed can effectively achieve good desalination results, high yield, and high product purity in large-scale production, further confirming its effectiveness and broad application prospects.

### 2.4. Comparison Between Traditional Methods and Crystallization-Based Techniques for GABA Purification

**Figure 3:**
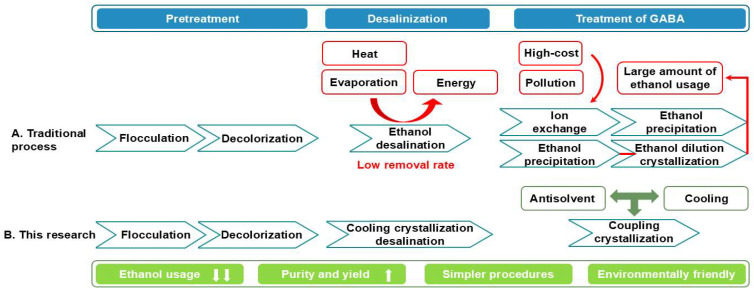
Flow chart for the traditional GABA purification processes [21,22,23] and this study.

Figure 3 presents the process flow of both the traditional GABA purification process and the novel process developed in this study. Table 3 compares their parameters. In the new process, the fermentation broth was initially subjected to flocculation and decolorization treatments. Subsequent to efficient cooling crystallization, we achieved a high removal efficiency while maintaining minimal loss of GABA. A comparative study of desalination effects between cooling crystallization and ethanol desalination was conducted using simulated fermentation broth. The experimental results indicated that after two desalination steps, the cooling crystallization removal efficiency reached 90.79%, significantly exceeding the 75% achieved by ethanol desalination. Additionally, this approach eliminates the requirement for ethanol and simplifies the desalination process, leading to a reduction in energy consumption. GABA with a purity of 98.66% was obtained through a “antisolvent–cooling” crystallization process in 3.2 L system. The overall GABA yield for the entire process was 67.32%, which surpasses that of other existing purification techniques. This new process markedly decreased ethanol usage, streamlined purification steps, and improved the environmental friendliness. Moreover, the new process offers notable advantages in operational simplicity, cost-effectiveness, scalability, and economic viability.

### 2.5. Techno-Economic Analysis

**Figure 4:**
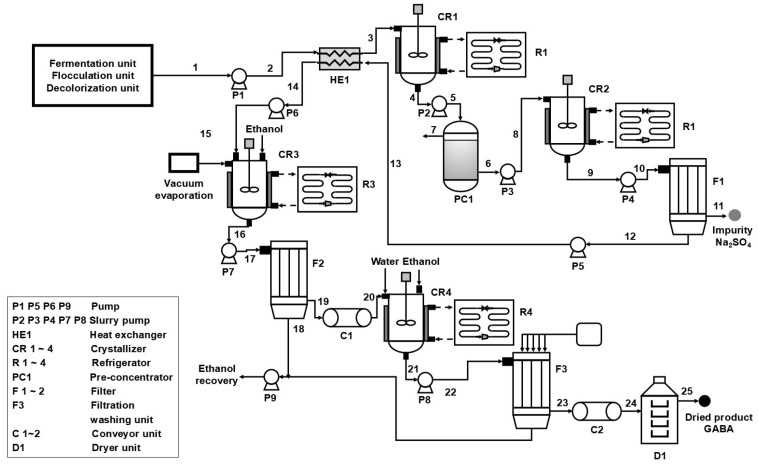
Industrial production model of GABA.

Based on previous experimental results, this study developed an integrated process flow for the production of high-purity GABA via fermentation. The process flow was evaluated by techno-economic analysis to confirm its feasibility and economic efficiency. As shown in Figure 4, the integrated process comprises fermentation, flocculation, decolorization, desalination, and GABA crystallization units, with detailed views provided for the desalination and GABA crystallization units. The fermentation broth is initially flocculated and decolorized, then pumped by P1 to heat exchanger HE1 for pre-cooling. The cold broth from HE1 is then directed to crystallizer CR1 for desalination via cooling crystallization. The required cooling duty is provided by the refrigeration unit R1. The crystallized slurry is pumped by P2 to PC1 for concentration. Then, it is sent to crystallizer CR2 for a secondary cooling crystallization. The condensate from PC1 (flow 7) can be reused as coolant for GABA crystallizers CR3 and CR4. The slurry exiting from the bottom of crystallizer CR2 is sent to filtration unit F1. In F1, vacuum filtration is employed to separate crystals from the residual liquid. The fermentation broth discharged from F1 is reintroduced into HE1 as a flow stream and subsequently pumped by P6 to the GABA crystallizer CR3 for concentration and the “antisolvent–cooling” crystallization process. The resulting slurry enters filtration unit F2, where GABA crystals are separated from the waste ethanol solution by vacuum filtration. Subsequently, the GABA crystals are conveyed by C1 to GABA crystallizer CR4 for dissolution and recrystallization. The crystallized slurry is then filtered and washed in F3, and the GABA crystals are conveyed by C2 to dryer D1. The dried GABA is collected as the final product. The waste ethanol solutions from F2 and F3 are combined and sent to an ethanol recovery unit for recycling.

This study employed optimized operational data from a 3.2 L system to construct a material balance model (Table S6). The “study estimate” method [26] is applied for production economics analysis, assessing both the cost-effectiveness and profit potential of the integrated process. The basic data and assumptions used in the cost estimation process are outlined in Table S7. The total capital investment (TCI) necessitated for the design, construction, and startup of the plant (Table S8), as well as the working capital requirements (Table S9) and the economic viability analysis for the plant, are summarized in Table 4. The profitability of the integrated process is assessed by integrating capital costs, operational expenses, and sales revenues. Overall, a plant with an annual GABA production capacity of 74.16 tons is projected to have a payback period of 1.98 years, a return on investment of 50.63%, and a break-even point of 48.65%, demonstrating the significant economic viability of the integrated process.

Compared to the traditional ethanol desalination process, the process proposed by Zhang et al. [23] has an estimated annual production cost (excluding labor, benefits, maintenance, and depreciation) of USD 641,000. In contrast, the process employed in this study has an annual working capital expenditure of USD 531,776.22, which is significantly lower. Furthermore, the equipment costs in this study amount to USD 486,844.46, which is also substantially less than the USD 666,812.65 estimated by Zhang et al. [23]. This cost reduction is primarily due to the relative simplicity of our crystallization process equipment and the streamlined process. However, the cooling crystallization process still faces some inherent challenges, such as providing sufficient cooling capacity in the summer and potential pipe crystallization issues during material and liquid transfer. These issues will be the focus of future research. Given the high added value of GABA products and the simplicity and environmental friendliness of the process, this project holds significant development potential and warrants further investment and implementation.

## 3. Materials and Methods

### 3.1. Materials

The fermentation strain utilized in this study was previously isolated from pickled vegetables in our laboratory [27], which was named Lb. CE701. Monosodium glutamate (MSG, purity ≥ 99%) was sourced from Henan Lianhua Monosodium Glutamate Co., Ltd. (Xiangcheng, China). Additional reagents such as Dansulfonyl chloride (HPLC, ≥98%), sodium acetate (GR, ≥99%), sodium bicarbonate (GR, ≥99.8%), γ-Aminobutyric acid (≥99%), and sodium sulfate (GR, ≥99.8%) were sourced from Shanghai Aladdin Reagent Co., Ltd. (Shanghai, China) Anhydrous ethanol was provided by Tianjin Jiangtian Chemical Co., Ltd. (Tianjin, China). All solutions were prepared using deionized water to ensure purity. The experiments for crystallization were carried out utilizing a thermostatic chamber (First Europe Technology Co., Ltd., Nanjing, China) equipped with a constant speed program.

All experiments detailed in this manuscript and its Supplementary Materials were performed at least three times to ensure the reproducibility and reliability of the findings.

### 3.2. Fermentation Processes and Pretreatment Methods

The fermentation process in this study employed the previously optimized laboratory conditions [28], and the fermentation broth underwent pretreatment involving flocculation and decolorization. Flocculation: An 800 mL aliquot of fermentation broth was introduced into a round-bottomed flask and maintained at 80 °C with a stirring speed of 300 rpm for 30 min. Diatomaceous earth was added to facilitate filtration. Decolorization: The pH of the filtrate obtained post-flocculation was adjusted to 5, and 2.5% (*w*/*v*) activated carbon was introduced. The mixture was then held at 70 °C and 300 rpm for 1 h before being filtered. The obtained filtrate was concentrated, and 1% (*w*/*v*) activated carbon was added under the same conditions for a second decolorization step.

### 3.3. Cooling Crystallization of GABA-Na_2_SO_4_ Solution and Actual Fermentation Broth

For the desalination process of the GABA-Na_2_SO_4_ solution, the experimental device depicted in Figure S1 was used to carry out the cooling crystallization experiment. Initially, a simulated fermentation solution of 800 mL was prepared, with mass concentrations of GABA and Na_2_SO_4_ at 110.9 g/L and 76.4 g/L, respectively, which were consistent with the actual fermentation solution. The solution was concentrated under vacuum (relative vacuum pressure (−0.1 MPa); unless otherwise specified, vacuum evaporation in this paper refers to that conducted at −0.1 MPa) at 60 °C until crystals occurred, then transferred to the crystallizer and cooled at a rate of 10 min/°C. When the temperature reached 12 °C (Table S5), 0.6 g of Na_2_SO_4_ seed crystals were added and cultured for 80 min, and the solution was further cooled at a controlled rate of (16 to 24 min/°C) to the experimental temperature (0 to −5 °C). Upon reaching the target temperature, the solution was maintained at this temperature for a specified duration (1 to 6 h) to promote crystal growth and precipitation, which is defined as the crystallization time. The solution was then filtered, the filtrate was proceeded to the next step, and the solid was dried for analysis. For the actual fermentation broth treatment, 800 mL of pretreated fermentation broth underwent the same desalination procedure.

### 3.4. Response Surface Optimization (RSM) for Cooling Crystallization Desalination

The most influential parameters were identified based on the results of the single-factor experiments (Figure S3). The RSM experimental factors included crystallization terminal temperature (−5 to −1 °C), crystallization time (2 to 6 h), and stirring rate (160 to 600 rpm), with the removal efficiency as the response variable. These parameters were analyzed and optimized using a three-factor, three-level central composite design (CCD). Detailed information about the experimental factors and levels is provided in Table S1. The design and results for the RSM analysis experiment are provided in Table S2. Data analysis was conducted using Design Expert software (version 13). The optimal conditions for desalination were determined through three principal analytical procedures: ANOVA, regression analysis, and response surface.

### 3.5. Comparison with Ethanol Desalination Method

An 800 mL simulated fermentation solution was prepared as described in Section 3.3. The solution was then concentrated under vacuum at 60 °C until crystals occurred. Ethanol desalination was performed according to the following method pioneered by Li et al. [21]: The concentrated solution was desalinated by the addition of 70% (*v*/*v*) anhydrous ethanol and set aside at room temperature for 1 h. After filtration, the filtrate was removed by vacuum rotary evaporation at 60 °C, and the solid was dried for analysis. Cooling crystallization was carried out as described in Section 3.3.

### 3.6. Optimization of the Process Conditions of “Antisolvent–Cooling” Crystallization for GABA

The actual fermentation broth (150 mL) after desalination treatment was concentrated under vacuum at 65 °C until crystals occurred. Then, concentration was stopped. The concentrated solution was then allowed to crystallize at 65 °C for 1 h, followed by the addition of anhydrous ethanol while cooling was initiated. The effects of various parameters on the GABA crystallization process were investigated, including ethanol addition ratio (0:1, 1:1, 2:1, 3:1, and 4:1), ethanol addition rate (10~30 mL/h), crystallization terminal temperature (5~20 °C), and crystallization time (0~4 h). The collected GABA crystals were washed with 50 mL of 95% (*v*/*v*) ethanol and then dried, followed by analysis of the resulting product. Once a condition was optimized, all subsequent experiments were conducted using the optimized condition.

### 3.7. Enlargement of the Crystallization Method to a 3.2 L System

To evaluate the effectiveness of the crystallization purification process after scale-up, the process was extended to a 3.2 L system. The cooling crystallization desalination was assessed by simulated fermentation solution, while the “Antisolvent–Cooling” crystallization process was evaluated by pre-treated and desalted actual fermentation broth. All experimental conditions were optimized.

### 3.8. Analytical Method

GABA samples underwent derivatization and were then analyzed via HPLC (Series 1200, Agilent, Santa Clara, CA, USA), as previously outlined [29]. A Hypersil ODS2 C18 (250 mm × 4.6 mm) column was used.

Calculation of removal efficiency: In the desalination experiment involving the GABA-Na_2_SO_4_ solution, the solid obtained from cooling crystallization was dried and weighed, and the GABA content could be measured. In this study, the removal efficiency is defined as the ratio of the mass of Na_2_SO_4_ removed from the solution during the cooling crystallization process to the initial mass of Na_2_SO_4_ in the solution. The formula is as follows:

Removal efficiency (%) = (m_1_ × (1 − ω_GABA_))/m_0_ × 100% (2)

where m_0_ is the mass of Na_2_SO_4_ added to the GABA-Na_2_SO_4_ solution (g), m_1_ is the mass of the solid obtained from cooling crystallization (g), and ω_GABA_ is the GABA mass fraction.

## 4. Conclusions

In summary, this study successfully developed a crystallization-based purification process. The process integrated cooling crystallization for desalination and a coupled “antisolvent–cooling” crystallization for GABA production. Through experimental optimization, we identified the optimal conditions for desalination and GABA crystallization, achieving a high purity of 98.73% and a yield of 64.36% in GABA production. Upon scaling up to a 3.2 L system, the yield of GABA reached 67.32%, with a purity of 98.66%, indicating the strong scalability of our crystallization process. Moreover, the process reduced ethanol usage compared to conventional methods, leading to decreased energy consumption, streamlined purification steps, and minimized environmental impact. Techno-economic analysis further confirmed the economic viability of the process, with a projected payback period of 1.98 years and a break-even point of 48.65% for a plant producing 74.16 tons of GABA annually. The findings from both process and economic analyses robustly establish the technical and economic viability of GABA production through fermentation. Thus, this study offers an innovative GABA purification method and lays a solid foundation for its industrial-scale production and application.

## Figures and Tables

**Table 1:** The ANOVA of CCD ^a^.

Source	Sum of Squares	Degree of Freedom	Mean Square	F Value	*p* Value
Model	1036.4	9	115.16	40.80	<0.0001
A	155.55	1	155.55	55.11	<0.0001
B	5.39	1	5.39	1.91	0.1972
C	56.79	1	56.79	20.12	0.0012
AB	1.28	1	1.28	0.4535	0.5159
AC	1.19	1	1.19	0.4201	0.5315
BC	0.98	1	0.98	0.3472	0.5688
A2	197.80	1	197.80	70.08	<0.0001
B2	40.36	1	40.36	14.30	0.0036
C2	13.38	1	13.38	4.74	0.0545
Residual	28.23	10	2.82		
Lack of Fit	15.34	5	3.07	1.19	0.4267
Pure Error	12.89	5	2.58		
Cor Total	1064.63	19			

^a^ R^2^ = 0.9735, adjusted R^2^ = 0.9496, predicted R^2^ = 0.8723.

**Table 2:** Results comparison.

Simulated Fermentation Solution	Removal Efficiency/%	Actual Fermentation Broth	Yield of GABA/%	Purity of GABA/%
800 mL system	90.79	800 mL system	64.36	98.73
3.2 L system	92.58	3.2 L system	67.32	98.66

**Table 3:** Parameter comparison for the traditional GABA purification processes [21,22,23] and this study.

Stages	Methods	Removal Efficiency/%	GABA Loss Rate ^a^/%	Purity of GABA /%	Yield of GABA /%	Ethanol Dosage ^b^/%	Process Steps
Desalination	Cooling crystallization	90.79	<5	-	-	0	1
	Ethanol desalination	75	10~20	-	-	230	2
Treatment of GABA	Coupled crystallization	-	-	98.66	67.32	300	2
	Ion Exchange [21]	-	-	98.66	50	unknow	4
	Ethanol Precipitation [23]	-	-	98.69	60	>900	4

a: GABA loss rate based on mass of removed Na_2_SO_4_; b: Ethanol dosage based on volume of liquid treated.

**Table 4:** Economic viability of GABA production plant.

Item	Value
Annual production/t	74.16
Annual sales revenue/USD	828,183.29
Total capital investment/USD	585,395.60
Total working capital/USD	531,776.22
Payback period/years	1.98
Return on capital investment/%	50.63
Break-even point/%	48.65

## Data Availability

All data, tables, and figures are originals.

## Supplementary Materials

The following supporting information can be downloaded at https://www.mdpi.com/article/10.3390/molecules30040897/s1: Figure S1: The diagram of the crystallization device [30]; Figure S2: Measurement of solubility. (a) Solubility of GABA in water; (b) Solubility of Na_2_SO_4_ in both pure water and aqueous solutions containing varying concentrations of GABA from the fermentation process; Figure S3: Influence of different cooling crystallization parameters on removal efficiency. (a) Crystallization terminal temperature; (b) Crystallization time; (c) Stirring rate; (d) Cooling rate; (e) Process duration; Table S1: The design and response of the CCD experimental; Table S2: Experimental and theoretical values of mole fraction of Na_2_SO_4_ [31]; Table S3: Van ‘t Hoff equation parameters of Na_2_SO_4_ at different concentrations of GABA; Table S4: The factors and levels from the RSM analysis experiment; Table S5: Solubility data of Na_2_SO_4_ in 220.8 g/L GABA solution; Table S6: Specifications and parameters for material balance; Table S7: Foundations and assumptions for cost estimation; Table S8: Estimates of total capital investment (TCI); Table S9: Estimates of working capital.
