# Supplementary material for: Development and Techno-Economic Evaluation of Crystallization Techniques for GABA Purification from Fermentation Broth
Source: Molecules. 2025 Feb 14;30(4):897. doi: 10.3390/molecules30040897 (PMC11858641; doi:10.3390/molecules30040897)
Supplement: Supplementary file 1 [file molecules-30-00897-s001.zip › molecules-3410029-supplementary.pdf]

Supporting information

Development and techno-economic evaluation of crystallization techniques for GABA Purification from fermentation broth

Yu Jing [1,†], Jinxu Zhang [1,†], Shengping You [1,*], Mengfan Wang [2], Rongxin Su [1,3] and Wei Qi [1,3]

[1] Chemical Engineering Research Center, School of Chemical Engineering and Technology, State Key
Laboratory of Chemical Engineering, Tianjin University, Tianjin 300350, China; jy_waldeinsamkeit@163.com (Y.J.); 13582364360@163.com (J.Z.); surx@tju.edu.cn (R.S.); qiwei@tju.edu.cn (W.Q.)
[2] School of Life Sciences, Tianjin University, 92 Weijin Road, Nankai District, Tianjin 300072, China; mwang@tju.edu.cn
[3] State Key Laboratory of Chemical Engineering, Tianjin University, Tianjin 300072, China
* Correspondence: ysp@tju.edu.cn
† These authors contributed equally to this work.

1 Experimental section

1.1 Measurement of Solubility

In this research, the solubility was determined by the static equilibrium method [1]. We evaluated the solubility of GABA in water and examined the solubility of Na_2SO_4 in solution at different concentrations of GABA. A specified volume of water was introduced into a conical flask, followed by an excess quantity of GABA. The mixture was maintained at a stable temperature and stirred for 8 h to achieve solid-liquid equilibrium. Then the solution was allowed to stand for 1 hour to facilitate the precipitation of undissolved solids. The supernatant was then rapidly filtered through a 0.45 μm filter membrane to measure the concentration of GABA. The solute composition was analyzed and calculated to obtain the solubility data of GABA. Solutions with GABA concentrations that were equivalent to 1, 2, 3, and 4 times that found in actual fermentation broth were prepared in conical flasks, followed by an excess quantity of Na_2SO_4. Following similar procedures as previously described allowed us to gather solubility information for Na_2SO_4 under diverse temperatures and various levels of GABA concentration. The SO_4^{2-} contents were measured by the gravimetric method with excess barium chloride solution [2].

1.2 Investigation of Cooling Crystallization Experimental Conditions

An 800 mL GABA-Na_2SO_4 simulated fermentation solution was prepared, with mass concentrations of GABA and Na_2SO_4 at 110.9 g/L and 76.4 g/L, respectively, consistent with the actual fermentation broth. The solution was vacuum concentrated at 60 °C until crystals occurred, and the concentrations of GABA and Na_2SO_4 in the solution at this point were measured. A solution with this GABA concentration was prepared, and the solubility of Na_2SO_4 in the solution at 10, 15, and 20 °C was measured according to step 1.1. The concentrated solution was cooled from 20 °C at a cooling rate of 10 min/°C to investigate the appropriate temperature for seed addition.

1.3 Single-factor Experiment

This study examined the effects of five factors on desalinization rate: crystallization terminal temperature (-5 ~ 0 °C), crystallization time (1 ~ 6 h), stirring rate (0 ~ 600 r/min), the process duration (60 ~ 120 min, refers to the time allowed for crystal growth following the addition of seed crystals) and cooling rate (16 ~ 24 min/°C) after the addition of crystal seeds. Once a condition was optimized, all subsequent experiments were conducted using the optimized condition.

1.3 Cooling Crystallization Device

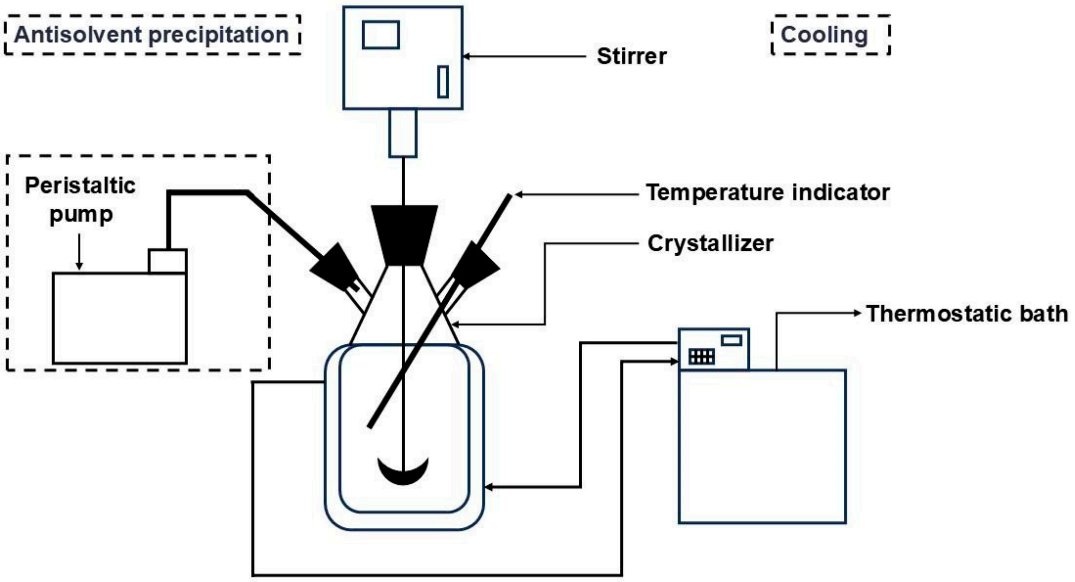

Figure S1. The diagram of the crystallization device.

1.4 Response Surface Optimization (RSM) for Cooling Crystallization Desalination

Table S1. The factors and levels of the CCD experimental.

Encoding	A:Crystallization terminal temperature（℃）	B:Stirring rate（r/min）	C:Crystallization time（h）
-1	-1	160	2
0	-3	380	4
1	-5	600	6

Table S2. The design and results for the RSM analysis experiment.

Trials	A:Crystallization end temperature (°C)	B:Stirring rate (r/min)	C:Crystallization time (h)	Removal efficiency (%)
1	0	0	0	70.63
2	0	0	0	72.50
3	1	-1	1	54.17
4	0	0	0	75.17
5	-1	1	0	66.20
6	-1	-1	1	63.20
7	-1	1	1	65.66
8	0	0	1	73.25
9	1	1	1	56.63
10	1	0	0	59.80
11	0	0	0	71.50
12	0	0	0	73.56
13	-1	1	-1	61.02
14	-1	-1	-1	58.36
15	0	1	0	67.80
16	0	0	0	73.26
17	1	-1	-1	52.47
18	0	-1	0	67.50
19	1	1	-1	51.93
20	0	0	-1	65.30

2 Results and Discussion

2.1 Analysis of Solubility

In comparison to the pure Na_2SO_4 solution, the mixed solution containing GABA is expected to influence the solubility of Na_2SO_4. Investigating this correlation is crucial for assessing the feasibility of employing cooling crystallization method to remove Na_2SO_4 from fermentation broth. Therefore, we first measured the solubility of GABA in water within a temperature range of 268.15 to 288.15 K, alongside determining the solubility of Na_2SO_4 at varying concentrations of GABA. As illustrates in Figure S2(a), GABA exhibits an exceptionally high solubility of 80 g/100 mL water at a low temperature of 268.15 K. Its solubility decreases as temperature drops further. Given that the concentration of GABA in fermentation broth was established at 11.09 g/100 mL water, it can be inferred that GABA would not occur under experimental conditions. Figure S2(b) shows the solubility of Na_2SO_4 in water across different temperatures and GABA concentrations. The solubility of Na_2SO_4 decreased with a reduction in temperature. Notably, at the same temperature, an increase in GABA concentration leads to a notable reduction in overall Na_2SO_4 solubility, indicating that GABA inhibits its dissolution process.

The experimental solubility data of Na_2SO_4 were analyzed by the Apelblat equation and Van't Hoff equation, respectively, resulting in a satisfactory fit (Table S).

Based on the Van't Hoff model, Gibbs free energy of dissolution and entropy of dissolution of Na_2SO_4 dissolved in different concentrations of GABA solutions were estimated (Table S3). Both enthalpy and Gibbs free energy of dissolution were found to be positive, indicating that the dissolution process of Na_2SO_4 in water is endothermic and non-spontaneous. This behavior is similarly observed in GABA solutions. Consequently, GABA does not alter the trend of Na_2SO_4 solubility with temperature, which was consistent with previous experimental findings. Additionally, it was observed that values of all the dissolution enthalpy surpasses dissolution entropy, implying that enthalpy acts as the main driving force during the dissolution of Na_2SO_4. The elevation in GABA concentration leads to a reduction in the enthalpy and entropy of dissolution, however, the change in Gibbs free energy associated with dissolution increases as the concentration of GABA rises. In conclusion, there is no obvious phase transition relationship between GABA and Na_2SO_4, therefore, effective separation can be achieved through cooling crystallization.

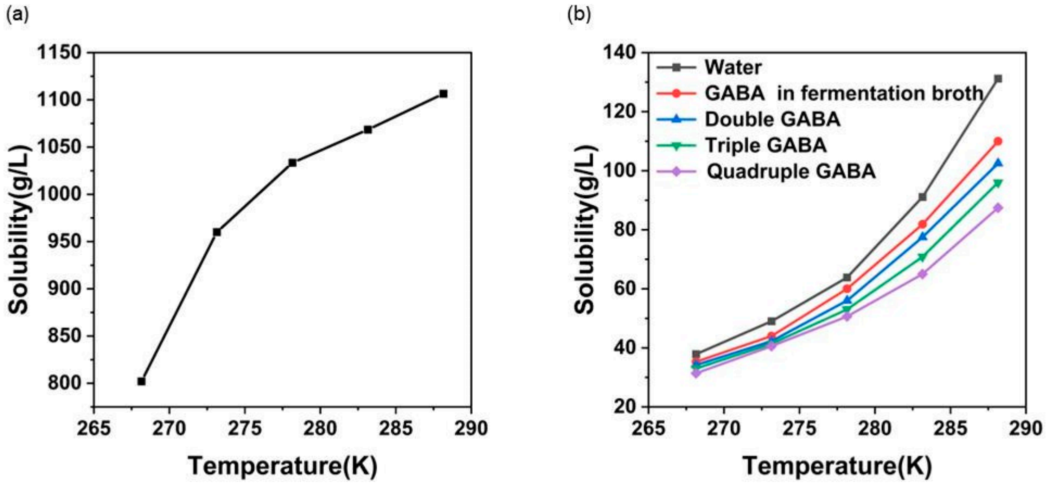

Figure S2. Measurement of solubility: (**a**) Solubility of GABA in water; (**b**) Solubility of Na_2SO_4 in both pure water and aqueous solutions containing varying concentrations of GABA from the fermentation process.

Table S3. Experimental and theoretical values of mole fraction of Na_2SO_4.

T /K	10^2X^{exp}	Apelblat model		Van't Hoff model	
		10^2X^{cal}	RD /%	10^2X^{cal}	RD /%
0% GABA					
268.15	0.4779	0.4334	9.3056	0.4265	10.7508
273.15	0.6173	0.6063	1.7786	0.6051	1.9722
278.15	0.8026	0.8439	-5.1455	0.8478	-5.6307
283.15	1.1417	1.1688	-2.3772	1.1738	-2.8071
288.15	1.6359	1.6112	1.5079	1.6068	1.7765
10^4*RMSD*		3.2089		3.6611	
10^2ARDP		4.0229		4.5874	
Single GABA concentration in fermentation broth					
268.15	0.4362	0.4127	5.3868	0.4068	6.7224
273.15	0.5433	0.5574	-2.5936	0.5570	-2.5262
278.15	0.7394	0.7499	-1.4290	0.7541	-1.9859
283.15	1.0061	1.0053	0.0810	1.0100	-0.3896
288.15	1.3473	1.3425	0.3551	1.3392	0.6051
10^4*RMSD*		1.3311		1.6404	
10^2ARDP		1.9691		2.4458	
Double GABA concentration in fermentation broth					
268.15	0.4145	0.3901	5.8786	0.3846	7.2113
273.15	0.5116	0.5224	-2.1134	0.5220	-2.0315
278.15	0.6767	0.6971	-3.0252	0.7008	-3.5675
283.15	0.9340	0.9270	0.7508	0.9311	0.3120
288.15	1.2316	1.2285	0.2564	1.2249	0.5395
10^4*RMSD*		1.5419		1.8093	
10^2ARDP		2.4049		2.7324	
Triple GABA concentration in fermentation broth					
268.15	0.3919	0.3683	6.0285	0.3645	6.9837
273.15	0.4914	0.4880	0.6787	0.4895	0.3788
278.15	0.6281	0.6446	-2.6367	0.6504	-3.5506
283.15	0.8376	0.8489	-1.3492	0.8556	-2.1469
288.15	1.1319	1.1143	1.5570	1.1146	1.5156
10^4*RMSD*		1.6012		1.9326	
10^2ARDP		2.4501		2.9151	
Quadruple GABA concentration in fermentation broth					
268.15	0.3658	0.3562	2.6371	0.3515	3.9043
273.15	0.4722	0.4626	2.0435	0.4625	2.0550
278.15	0.5893	0.5993	-1.6939	0.6026	-2.2571
283.15	0.7534	0.7744	-2.7819	0.7777	-3.2278
288.15	1.0124	0.9982	1.4101	0.9949	1.7278
10^4*RMSD*		1.3631		1.6568	
10^2ARDP		2.1133		2.6344	

Table S4. Van't Hoff equation parameters of Na_2SO_4 at different concentrations of GABA.

GABA	0	1	2	3	4
ΔH_d(kJ·mol^{-1})	42.6043	38.2662	37.2124	35.9041	33.4165
ΔG_d (kJmol^{-1})	2.3125	2.5438	2.5438	2.7751	3.0063
ΔS_d(J·mol^{-1} ·K^{-1})	144.8563	128.4285	124.6399	119.1047	109.3302

2.2 Determination of experimental conditions for cooling crystallization

For the seeded crystallization process, the concentration of the solution prior to crystallization and the temperature at which seeds are added to induce crystallization are crucial. By vacuum concentration, the mass concentrations of GABA and Na_2SO_4 in the solution reached 220.8 g/L and 151.6 g/L, respectively. The solubility of Na_2SO_4 at this GABA concentration is shown in Table S5. Although the solution had reached supersaturation at 15°C, no crystal formation was observed after adding seeds. However, when the temperature was lowered to 12°C, a significant amount of crystals precipitated after adding seeds. Therefore, we chose to add the seed crystals at 12°C for crystallization.

Table S5. Solubility data of Na_2SO_4 in 220.8 g/L GABA solution.

Solubility/g/L	10°C	15°C	20°C
Na_2SO_4	85.2	130.6	175.3

2.3 Single-factor Experiment

This study assessed the impact of various operational conditions on cooling crystallization desalination, and the results are depicted in Figure S3. Contrary to expectations based on Na_2SO_4 solubility trend, lower crystallization terminal temperature did not achieve higher removal efficiency. As shown in Figure S3(a), when temperatures drop below -3 °C, the increase in removal efficiency diminishes and may even decline. This can be attributed to the reduction in thermal motion of solute molecules at lower temperatures, which decreases their molecular collisions and nucleation. Additionally, at low temperatures, the saturation temperature for supersaturation is also reduced, and as solutes crystallize, their concentration decreases, making nucleation more difficult. The solution attains its freezing point at -5°C, the solution freezes, further reducing removal efficiency. Thus, -3°C was determined as the optimal terminal temperature. The impact of the crystallization time was examined (Figure S3(b)). As anticipated, extending the time for crystal growth correlated with an increased removal efficiency. However, removal efficiency did not further increase once mass transfer equilibrium was attained. Considering both time efficiency and energy usage, this study identified a crystallization time of 4 hours as optimal. During the crystallization process, stirring plays a crucial role in enhancing the heat and mass transfer efficiency of the solution. A low stirring rate may lead to non-uniform mixing, resulting in excessively high local supersaturation. Conversely, a high stirring rate generates strong shear forces that can inhibit crystal growth. Therefore, on the basis of achieving effective mass and heat transfer, a relatively lower stirring rate is recommended. In this study, we selected a stirring rate of 380

r/min (Figure S3(c)). Compared with other factors, the cooling rate and crystal cultivation time after the addition of crystal seeds have a relatively minor influence on the removal efficiency (Figure S3(d) and (e)). These parameters mainly affect the particle size of the final product. For this study, a process duration of 80 minutes and a cooling rate of 20 min/°C were selected.

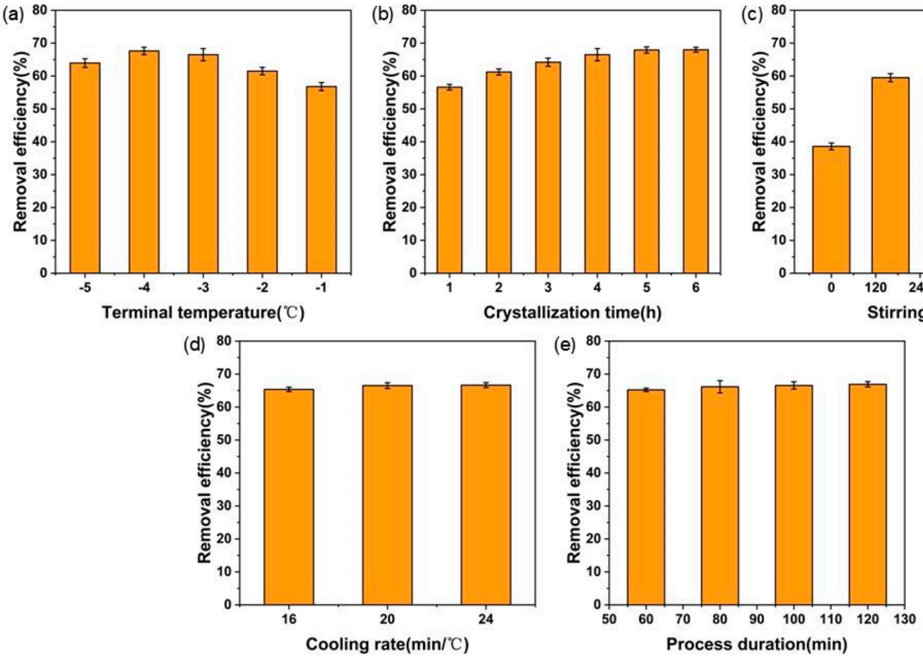

Figure S3. Influence of different cooling crystallization parameters on removal efficiency: (**a**) Crystallization terminal temperature; (**b**) Crystallization time; (**c**) Stirring rate; (**d**) Cooling rate; (**e**) Process duration.

3 Other tables

Table S6. Specifications and parameters for material balance.

Parameter	Value
Feed mass flow rate (m^3/year)	955259
Antisolvent mass flow rate 1 (m^3/year)	349800
Antisolvent mass flow rate 2 (m^3/year)	111300
Waste ethanol mass flow rate (m^3/year)	572400
Product composition	GABA (98.66%)
Product mass flow rate（TPA）	74160
Impurity composition	Na_2SO_4(93.2%)
Impurity mass flow rate（TPA）	65155

Table S7. Foundations and assumptions for cost estimation.

Foundations/Assumptions	Value, unit
Plant capacity	955259(m^3/year)
Shift and working hour per day	2 shifts, 16h
Design margin	10%
Economic life of the plant & equipment	15years
Construction period	1 year
Capacity utilization	75% for 1st year, 100% 2nd year onwards

Table S8. Estimates of total capital investment (TCI).

S. No.	Item	Cost（US$）
1	Civil Works	27918.8000
2	Plant and equipment	486844.4613
3	Furniture and fixtures, quality control equipment	6979.7000
4	Contingency	52174.29613
5	Margin money	11478.3460
6	Total capital investment (TCI):	585395.6034

Table S9. Estimates of working capital.

Item	Cost（US$）
Raw material	251772.5900
Utilities	33612.4800
Salaries and wages	209391.0000
Maintenance	5842.1300
Depreciation	31158.0160
Total working capital	531776.2160

4 References

1. Su, N.N.; Wang, Y.L.; Xiao, Y.; Lu, H.J.; Lou, Y.J.; Huang, J.J.; He, M.; Li, Y.; Hao, H.X. Mechanism of Influence of Organic Impurity on Crystallization of Sodium Sulfate. *Ind. Eng. Chem. Res.* **2018,** 57, 1705-1713.
2. *GB/T 13025.8-2012*; General Test Method in Salt Industry-Determination of Sulfate. General Administration of Quality Supervision,Inspection and Quarantine of the People's Republic of China, China Standards Press: Beijing, China, 2012.
